# Combination of remazolam and alfentanil on hemodynamics, postoperative cognitive function, and adverse cardiovascular events in elderly patients undergoing laparoscopic cholecystectomy: a randomized controlled trial

**DOI:** 10.3389/fphar.2025.1653593

**Published:** 2025-11-05

**Authors:** Chaolei Liu, Jin Li, Junna He, Jingjing Zhang, Lijun Bo, Jiaojiao Yang, Yuan Ma, Jianzhang Qin, Yanjie Yang, Zhangnan Sun

**Affiliations:** ^1^ Department of Anesthesiology, The Second Hospital of Hebei Medical University, Shijiazhuang, China; ^2^ Department of Neurology, The Second Hospital of Hebei Medical University, Shijiazhuang, China; ^3^ Department of Hepatobiliary Surgery, The Second Hospital of Hebei Medical University, Shijiazhuang, China

**Keywords:** remazolam, propofol, alfentanil, laparoscopic cholecystectomy, hemodynamics, postoperative cognitive dysfunction, postoperative delirium, adverse cardiovascular events

## Abstract

**Objective:**

To evaluate the effects of a remazolam-alfentanil combination versus a propofol-alfentanil combination on intraoperative hemodynamics, postoperative cognitive function, and cardiovascular adverse events in elderly patients undergoing laparoscopic cholecystectomy.

**Methods:**

This prospective, randomized controlled trial enrolled 116 elderly patients (aged 60–80 years, ASA I–III) undergoing laparoscopic cholecystectomy from January 2022 to June 2023. Patients were randomly assigned (1:1) to receive either remazolam-alfentanil (n = 58) or propofol-alfentanil (n = 58). Primary outcomes included intraoperative hemodynamic parameters (MAP, HR, CO, CI, SVR), cognitive function (MMSE and MoCA scores, and incidence of postoperative delirium [POD]), and incidence of cardiovascular events. Secondary outcomes included recovery times and postoperative VAS and Ramsay scores.

**Results:**

A total of 112 patients (56 per group) completed the study. The remazolam group had significantly shorter times to awakening (10.41 ± 2.09 vs. 12.68 ± 2.73 min), extubation (11.17 ± 2.11 vs. 14.34 ± 2.62 min), and anesthesia duration (22.53 ± 6.66 vs. 28.81 ± 7.05 min) (all P < 0.001). Intraoperatively, the remazolam group showed more stable hemodynamics with higher MAP, HR, CO, and CI (P < 0.05). Postoperative MMSE and MoCA scores were significantly higher on days 3 and 7 (P < 0.05). The incidence of POD at day 7 was significantly lower in the remazolam group (3.6% vs. 16.1%, P = 0.031). The incidence of cardiovascular events was lower in the remazolam group (21.4% vs. 71.4%, P < 0.001), particularly hypotension (7.1% vs. 62.5%) and bradycardia (14.3% vs. 53.6%). VAS and Ramsay scores were also lower at 6 and 12 h postoperatively (P < 0.001).

**Conclusion:**

The remazolam-alfentanil combination provided faster recovery, improved hemodynamic stability, better cognitive outcomes, and fewer cardiovascular events than propofol-alfentanil in elderly laparoscopic cholecystectomy patients. While promising, these findings warrant confirmation in larger, multicenter trials.

**Clinical Trial Registration:**

https://www.chictr.org.cn/showproj.html?proj=210389, Identifier ChiCTR2300077536.

## 1 Introduction

Laparoscopic cholecystectomy is a widely adopted minimally invasive surgical procedure for gallbladder diseases, offering benefits such as reduced trauma, less postoperative pain, and quicker recovery compared to open surgery (Kim and Donahue, 2018; Osborne et al., 2006). However, the choice of anesthetic regimen is crucial, especially in elderly patients who often present with comorbidities and age-related physiological changes (Araújo et al., 2019). These patients exhibit increased sensitivity to anesthetics, predisposing them to hemodynamic instability, impaired drug metabolism, and an elevated risk of postoperative cognitive dysfunction (POCD) (Vutskits and Xie, 2016; Berger et al., 2018). Therefore, optimizing anesthesia to mitigate these risks is paramount.

Propofol, a common intravenous anesthetic, is known for its rapid onset and recovery. However, it can cause significant dose-dependent hypotension and bradycardia, particularly in the elderly (Chan and Chung, 1996). Remazolam, an ultra-short-acting benzodiazepine, is metabolized by tissue esterases into an inactive metabolite, resulting in rapid offset of action and minimal accumulation, even with continuous infusion (Kilpatrick, 2021; Zhou et al., 2020). Studies suggest remazolam offers greater hemodynamic stability and a lower incidence of respiratory depression compared to propofol, making it a potentially safer option for elderly or hemodynamically compromised patients (Guo et al., 2022). Preclinical and clinical evidence also indicates remazolam may possess neuroprotective properties, potentially reducing inflammation and oxidative stress, and preserving cognitive function (Xie et al., 2021; Shi et al., 2022; Liu et al., 2021). Improved neuropsychiatric recovery has been observed with remazolam in endoscopic procedures (Rex et al., 2018; Pastis et al., 2019; Tan et al., 2022). A recent meta-analysis also suggests remazolam provides comparable sedation with better hemodynamic stability than propofol during gastrointestinal endoscopy (Barbosa et al., 2024).

Alfentanil, a short-acting opioid, is often used for its potent analgesic effects and rapid offset (Kramer et al., 1983). Combining alfentanil with a sedative aims to achieve balanced anesthesia with optimal hypnosis, analgesia, and hemodynamic control. While propofol-alfentanil is a common combination, the potential benefits of a remazolam-alfentanil regimen in elderly surgical patients, particularly concerning cognitive outcomes and hemodynamic stability, warrant further investigation.

Despite the growing interest in remazolam, there is a paucity of head-to-head randomized controlled trials directly comparing the remazolam-alfentanil combination with the standard propofol-alfentanil regimen, particularly within the context of laparoscopic cholecystectomy in the elderly. This represents a critical knowledge gap in clinical practice. Therefore, we hypothesized that the remazolam-alfentanil combination would be associated with faster anesthesia recovery, greater hemodynamic stability, better preservation of postoperative cognitive function, and a lower incidence of adverse events compared to the propofol-alfentanil combination in this patient population.

## 2 Patients and methods

### 2.1 Study design and ethical approval

This single-center, prospective, randomized, parallel-group controlled trial was conducted at The Second Hospital of Hebei Medical University, Shijiazhuang, China, from November 2023 to June 2024. The study protocol was approved by the Institutional Review Board of The Second Hospital of Hebei Medical University (Approval No: 2023-R321) and registered with the Chinese Clinical Trial Registry (ChiCTR2300077536). The trial was conducted in accordance with the Declaration of Helsinki and CONSORT 2010 guidelines (Schulz et al., 2010). Written informed consent was obtained from all participants or their legal guardians before enrollment.

### 2.2 Patient selection

#### 2.2.1 Inclusion criteria

Age 60–80 years. Definitive diagnosis of gallbladder disease (e.g., cholelithiasis, chronic cholecystitis, gallbladder polyps) requiring laparoscopic cholecystectomy. American Society of Anesthesiologists (ASA) physical status I–III (Hendrix and Garmon, 2025). Ability to provide informed consent and communicate effectively. Body Mass Index (BMI) 18.5–30 kg/m^2^.

#### 2.2.2 Exclusion criteria

Contraindications to general anesthesia or laparoscopic surgery. Pre-existing significant cognitive impairment (e.g., dementia, MMSE score <24 adjusted for education). Use of psychotropic medications within 3 months prior to surgery. Known allergy or hypersensitivity to study drugs (remazolam, propofol, alfentanil, cisatracurium). Severe uncompensated cardiovascular, respiratory, hepatic, or renal disease. History of alcohol or drug abuse. Emergency surgery or conversion to open cholecystectomy. Participation in another clinical trial within 30 days.

### 2.3 Randomization and blinding

Eligible patients were randomly assigned in a 1:1 ratio to either the experimental group (Remazolam group) or the control group (Propofol group). Randomization was performed using a computer-generated random number sequence, with allocation concealment maintained through sequentially numbered, opaque, sealed envelopes (SNOSE) prepared by an independent statistician not involved in patient recruitment or assessment. Patients, outcome assessors (for cognitive function and postoperative pain/sedation), and data analysts were blinded to group allocation. The attending anesthesiologists administering the study drugs were not blinded due to the nature of the interventions, but they were not involved in postoperative data collection or assessment. To maintain blinding for other personnel, study drugs were prepared in identical syringes by an anesthesiologist not involved in direct patient care or data collection during the surgery.

### 2.4 Anesthesia management

All patients fasted for at least 8 h for solids and 2 h for clear liquids before surgery. Upon arrival in the operating room, standard monitoring was initiated, including electrocardiogram (ECG), non-invasive blood pressure (NIBP), pulse oximetry (SpO_2_), and Bispectral Index (BIS) (Aspect Medical Systems, Newton, MA, United States). An intravenous line was established, and lactated Ringer’s solution was infused at 6–8 mL/kg/h.

Pre-oxygenation with 100% oxygen (6 L/min) was administered for 3 min via a face mask. Anesthesia induction commenced as follows:

• Experimental (Remazolam) Group: Received an initial bolus of remazolam besylate (Yichang Humanwell Pharmaceutical Co., Ltd., Yichang, China) 0.2 mg/kg intravenously.
• Control (Propofol) Group: Received an initial bolus of propofol (Fresenius Kabi Austria GmbH, Graz, Austria) 1.5 mg/kg intravenously.

If the BIS value did not decrease to 40–60 within 2 min, an additional dose of remazolam (0.05 mg/kg) or propofol (0.5 mg/kg) was administered. Once an adequate depth of anesthesia was achieved (loss of eyelash reflex and BIS 40–60), alfentanil 15 μg/kg (Yichang Humanwell Pharmaceutical Co., Ltd.) was administered intravenously to all patients, followed by cisatracurium besylate 0.15–0.2 mg/kg to facilitate tracheal intubation. Tracheal intubation was performed 3 min after cisatracurium administration. Mechanical ventilation was initiated and adjusted to maintain end-tidal carbon dioxide (EtCO_2_) between 35 and 45 mmHg.

Anesthesia was maintained with:

• Experimental (Remazolam) Group: Continuous infusion of remazolam at 0.5–1.0 mg/kg/h and alfentanil at 0.25–0.5 μg/kg/min, titrated to maintain BIS between 40 and 60.
• Control (Propofol) Group: Continuous infusion of propofol at 4–8 mg/kg/h and alfentanil at 0.25–0.5 μg/kg/min, titrated to maintain BIS between 40 and 60.

In both groups, anesthesia was supplemented with inhaled sevoflurane (0.5–1.0 MAC if needed, based on clinical signs and BIS) and intermittent boluses of cisatracurium to maintain adequate muscle relaxation. All infusions (remazolam/propofol and alfentanil) and sevoflurane were discontinued at the beginning of skin closure. Residual neuromuscular blockade was reversed with neostigmine 0.04 mg/kg and atropine 0.02 mg/kg if necessary. Patients were extubated when they were awake, responsive to commands, and demonstrated adequate spontaneous ventilation (tidal volume >5 mL/kg, respiratory rate 10–20 breaths/min, SpO_2_ >95% on room air or supplemental oxygen).

### 2.5 Outcome measures

#### 2.5.1 Primary outcomes

• Hemodynamic Parameters: Mean arterial pressure (MAP), heart rate (HR), cardiac output (CO), cardiac index (CI), systemic vascular resistance (SVR), and BIS were recorded using a non-invasive continuous hemodynamic monitoring system (TL-400, Zhejiang Sunview Biopharmaceutical Equipment Co., Ltd., Hangzhou, China) and a BIS monitor at the following time points: T0 (Baseline), T1 (1 min after induction), T2 (Immediately after intubation), T3 (1 min post-intubation), T4 (5 min post-intubation), T5 (At pneumoperitoneum), T6 (At gallbladder removal), T7 (At skin closure).
• Postoperative Cognitive Function: Assessed using the Mini-Mental State Examination (MMSE) (Folstein et al., 1975) and Montreal Cognitive Assessment (MoCA) (Nasreddine et al., 2005) at D0 (Preoperatively), D3 (Postoperative day 3), and D7 (Postoperative day 7). POCD was defined as a decrease of ≥1 standard deviation (SD) from the individual’s preoperative baseline score on either MMSE or MoCA, or a combined Z-score decrease of ≥1.96 (Evered et al., 2018). Postoperative delirium (POD) was assessed on postoperative days 1, 3, and 7 using the Confusion Assessment Method (CAM) (Inouye et al., 1990).
• Incidence of Adverse Cardiovascular Events: Monitored from anesthesia induction until discharge from the post-anesthesia care unit (PACU). Events included: Hypotension (MAP <65 mmHg or >30% decrease from baseline for >1 min, requiring intervention), Bradycardia (HR < 50 beats/min requiring intervention), Hypertension (MAP >110 mmHg or >30% increase from baseline for >1 min), Tachycardia (HR > 100 beats/min for >1 min), and Hypoxemia (SpO_2_ <90% for >1 min).

#### 2.5.2 Secondary outcomes

• Anesthesia Recovery Times: Time to awakening, time to extubation, duration of anesthesia, and length of PACU stay.
• Postoperative Analgesic and Sedative Effects: Pain intensity (VAS; 0–10) and sedation level (Ramsay Sedation Scale; 1–6) (Ramsay et al., 1974) at 2, 6, 12, and 24 h postoperatively.
• Other Outcomes: Incidence of postoperative nausea and vomiting (PONV) within 24 h, requirement for rescue analgesia, and total intraoperative alfentanil consumption.

### 2.6 Sample size calculation

The sample size was determined based on the primary outcome of MAP changes and postoperative cognitive function scores. Based on previous studies comparing novel sedatives with propofol (Guo et al., 2022; Tan et al., 2022), we anticipated a clinically meaningful difference in MAP (effect size d = 0.5) and MoCA scores (effect size d = 0.5). To detect such a difference with 80% power (1-β = 0.80) at a two-sided alpha level of 0.05, a minimum of 51 patients per group was required for continuous outcomes. To account for potential dropouts (approximately 10%–15%) and to ensure adequate power for comparing proportions of adverse events, we aimed to enroll 58 patients per group, totaling 116 patients.

### 2.7 Statistical analysis

Statistical analysis was performed using SPSS version 29.0 (IBM Corp., Armonk, NY, United States). Continuous variables were expressed as mean ± standard deviation (SD) and compared using independent samples t-tests after confirming normality with the Shapiro-Wilk test. Non-normally distributed data were compared using the Mann-Whitney U test. Categorical data were presented as frequency (percentage) and compared using the Chi-square test or Fisher’s exact test, as appropriate. Hemodynamic parameters and cognitive scores over time were analyzed using a two-way repeated measures analysis of variance (ANOVA) with group as a between-subject factor and time as a within-subject factor, followed by Bonferroni *post hoc* tests. A P-value <0.05 was considered statistically significant. A per-protocol analysis was performed for patients who completed the study. Given the low and non-differential dropout rate (two patients per group), the per-protocol analysis results were consistent with a sensitivity analysis performed using an intention-to-treat principle with last observation carried forward, confirming the robustness of the findings.

## 3 Results

### 3.1 Patient enrollment and baseline characteristics

**FIGURE 1:**
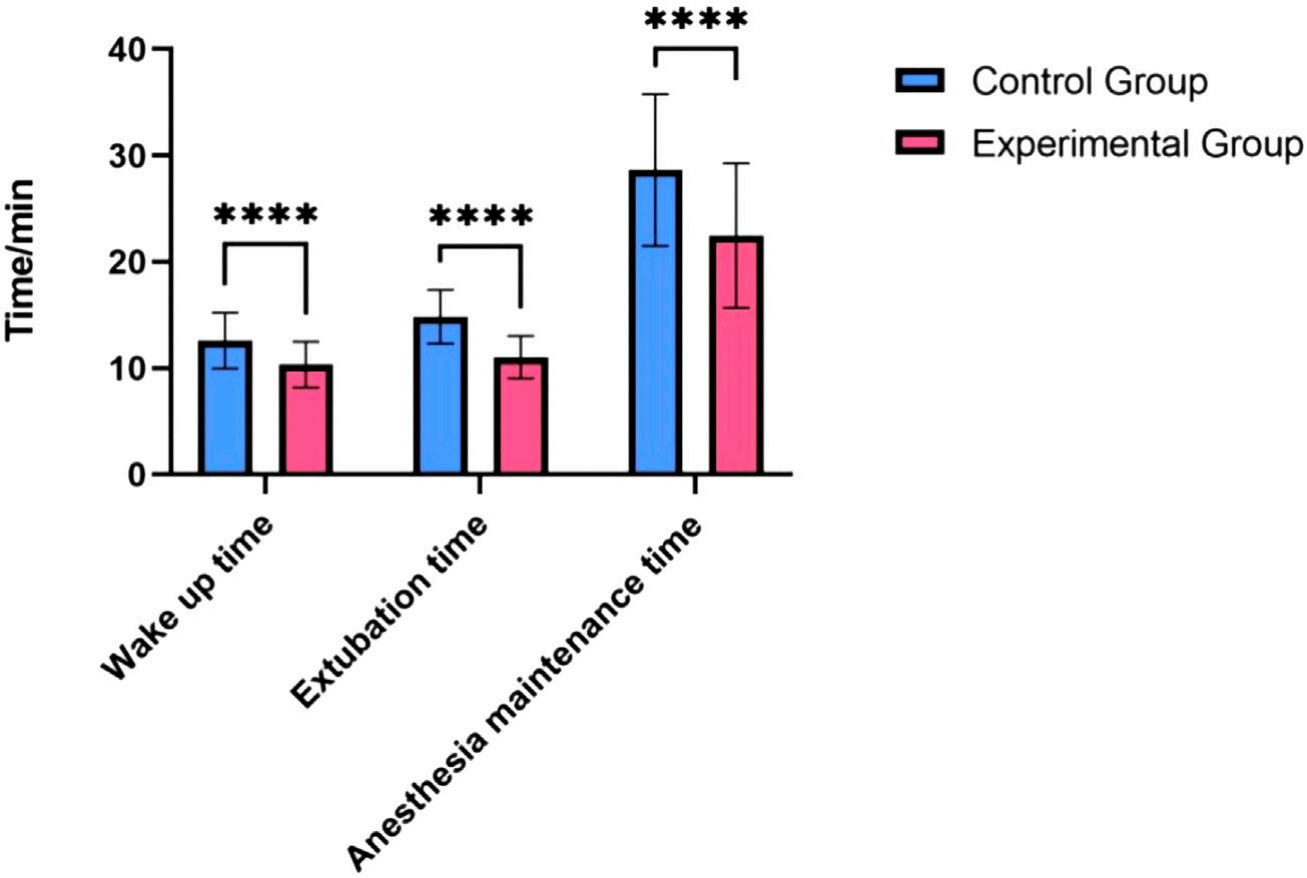
CONSORT (Consolidated Standards of Reporting Trials) flow diagram of patient enrollment, allocation, follow-up, and analysis. The diagram details the number of patients screened for eligibility, the reasons for exclusion, the number of patients randomized to the Remazolam and Propofol groups, and the number of patients included in the final per-protocol analysis.

From November 2023 to June 2024, 152 patients were assessed for eligibility. Of these, 36 did not meet inclusion criteria or declined to participate. Consequently, 116 patients were randomized: 58 to the Remazolam group and 58 to the Propofol group. Two patients in each group withdrew consent before surgery. Thus, 112 patients (56 per group) completed the study and were included in the final analysis (Figure 1). Baseline demographic, clinical, and laboratory characteristics were comparable between the two groups (Table 1).

**TABLE 1:** Baseline demographic and clinical characteristics of patients (n = 112).

Characteristic	Remazolam group (n = 56)	Propofol group (n = 56)	P-value
Age (years), mean ± SD	68.25 ± 5.46	69.45 ± 5.89	0.298
Sex (Male/Female), n (%)	30 (53.6%)/26 (46.4%)	32 (57.1%)/24 (42.9%)	0.705
Body Mass Index (kg/m^2^), mean ± SD	24.1 ± 2.5	24.5 ± 2.8	0.451
ASA Physical Status, n (%)			0.573
I	18 (32.1%)	15 (26.8%)	
II	30 (53.6%)	33 (58.9%)	
III	8 (14.3%)	8 (14.3%)	
Preoperative MMSE score, mean ± SD	28.5 ± 1.2	28.3 ± 1.4	0.460
Preoperative MoCA score, mean ± SD	27.9 ± 1.3	27.7 ± 1.5	0.488
Duration of Surgery (min), mean ± SD	55.8 ± 10.2	57.1 ± 9.8	0.512
Diagnosis, n (%)			0.815
Chronic Cholecystitis	11 (19.6%)	15 (26.8%)	
Gallbladder Stones	26 (46.4%)	24 (42.9%)	
Gallbladder Polyps	19 (33.9%)	17 (30.4%)	
Hypertension, n (%)	18 (32.1%)	20 (35.7%)	0.693
Diabetes Mellitus, n (%)	10 (17.9%)	12 (21.4%)	0.621
Coronary Artery Disease, n (%)	7 (12.5%)	9 (16.1%)	0.591
ALT (U/L), mean ± SD	25.4 ± 8.1	26.9 ± 9.2	0.435
AST (U/L), mean ± SD	23.8 ± 7.5	24.6 ± 8.0	0.617
Creatinine (μmol/L), mean ± SD	78.3 ± 15.2	80.1 ± 16.5	0.598

Data are presented as mean ± standard deviation or n (%). ASA, american society of anesthesiologists; MMSE, Mini-Mental State Examination; MoCA, montreal cognitive assessment; ALT, alanine aminotransferase; AST, Aspartate aminotransferase. P-values derived from t-test for continuous variables and Chi-square test or Fisher’s exact test for categorical variables.

### 3.2 Anesthesia recovery

Patients in the Remazolam group had significantly shorter anesthesia recovery times compared to the Propofol group. As detailed in Table 2, time to awakening (10.41 ± 2.09 min vs. 12.68 ± 2.73 min, P < 0.001), time to extubation (11.17 ± 2.11 min vs. 14.34 ± 2.62 min, P < 0.001), and duration of anesthesia maintenance (22.53 ± 6.66 min vs. 28.81 ± 7.05 min, P < 0.001) were all significantly reduced in the Remazolam group. Length of PACU stay was also shorter in the Remazolam group (35.2 ± 5.1 min vs. 42.8 ± 6.3 min, P < 0.001).

**TABLE 2:** Comparison of anesthesia recovery parameters.

Parameter (min)	Remazolam group (n = 56)	Propofol group (n = 56)	Mean difference [95% CI]	P-value
Time to awakening	10.41 ± 2.09	12.68 ± 2.73	−2.27 [-3.22, −1.32]	<0.001
Time to extubation	11.17 ± 2.11	14.34 ± 2.62	−3.17 [-4.14, −2.20]	<0.001
Duration of anesthesia	22.53 ± 6.66	28.81 ± 7.05	−6.28 [-9.21, −3.35]	<0.001
Length of PACU stay	35.2 ± 5.1	42.8 ± 6.3	−7.60 [-9.98, −5.22]	<0.001

Data are presented as mean ± standard deviation (min). CI, confidence interval; PACU, Post-Anesthesia Care Unit. P-values derived from independent samples t-test.

### 3.3 Hemodynamic parameters

Baseline hemodynamic parameters (T0) were comparable between the two groups (Table 3). Following induction and intubation (T1-T4), MAP and HR in the Propofol group showed a more pronounced decrease. The Remazolam group maintained significantly higher MAP and HR at T1, T2, T3, and T4 (all P < 0.001). Similarly, CO and CI were significantly higher in the Remazolam group at T1-T4 (all P < 0.001). SVR was significantly lower in the Propofol group at T1-T4 (all P < 0.001). Intraoperative BIS values were successfully maintained within the target range of 40–60 in both groups with no significant difference between them (P > 0.05). SpO_2_ levels remained stable. Hemodynamic parameters at T5-T7 also showed better stability in the Remazolam group.

**TABLE 3:** Comparison of intraoperative hemodynamic and anesthesia depth parameters (mean ± SD).

Parameter	Group	T0 (baseline)	T1 (post-induction)	T2 (post-intubation)	T3 (1 min post-Intub.)	T4 (5 min post-Intub.)
MAP (mmHg)	Remazolam	103.41 ± 8.63	99.32 ± 6.68*	101.27 ± 8.49*	98.95 ± 7.87*	99.34 ± 7.22*
	Propofol	104.82 ± 7.64	72.34 ± 6.42	87.25 ± 7.81	81.28 ± 7.46	82.32 ± 7.86
HR (bpm)	Remazolam	77.34 ± 9.28	76.81 ± 8.21*	77.01 ± 9.48*	75.94 ± 8.69*	76.17 ± 8.58*
	Propofol	76.58 ± 8.82	60.03 ± 9.20	62.79 ± 8.88	66.08 ± 8.85	64.75 ± 9.17
CO (L/min)	Remazolam	4.64 ± 0.73	4.24 ± 0.71*	4.32 ± 0.68*	4.20 ± 0.52*	4.28 ± 0.73*
	Propofol	4.78 ± 0.67	3.25 ± 0.69	3.68 ± 0.76	3.88 ± 0.66	3.59 ± 0.64
CI (L/min/m^2^)	Remazolam	3.49 ± 0.89	3.27 ± 0.75*	3.55 ± 0.45*	3.64 ± 0.24*	3.62 ± 0.48*
	Propofol	3.58 ± 0.90	2.71 ± 0.64	2.63 ± 0.77	2.83 ± 0.38	2.69 ± 0.21
SVR (dyn·s/cm^5^)	Remazolam	1192 ± 108	1225 ± 125*	1209 ± 130*	1162 ± 127*	1128 ± 124*
	Propofol	1185 ± 114	1060 ± 133	1015 ± 122	975 ± 129	949 ± 127
BIS	Remazolam	96.5 ± 2.1	49.8 ± 4.5	48.2 ± 3.9	47.5 ± 4.1	48.1 ± 3.8
	Propofol	97.1 ± 1.9	48.9 ± 5.0	47.9 ± 4.2	48.3 ± 3.6	48.8 ± 4.0

MAP: mean arterial pressure; HR, heart rate; CO, cardiac output; CI, cardiac index; SVR, systemic vascular resistance; BIS, Bispectral Index. Values are mean ± SD. *P < 0.001 Remazolam group vs. Propofol group at respective time points (T1-T4). No significant differences at T0 for any parameter or at any time point for BIS (P > 0.05).

### 3.4 Postoperative cognitive function

Preoperative MMSE and MoCA scores were similar between groups (P > 0.05) (Table 1). As shown in Table 4, on postoperative day 3 (D3) and day 7 (D7), patients in the Remazolam group had significantly higher MMSE and MoCA scores compared to the Propofol group (all P < 0.001). The incidence of POCD (defined as a decrease of ≥1 SD from an individual’s preoperative baseline score) at D3 was significantly lower in the Remazolam group (5.4% [3/56] vs. 19.6% [11/56], P = 0.025) and at D7 (3.6% [2/56] vs. 14.3% [8/56], P = 0.048). The cumulative incidence of postoperative delirium (POD) assessed by CAM by day 7 was also significantly lower in the Remazolam group (3.6% [2/56] vs. 16.1% [9/56], P = 0.031).

**TABLE 4:** Comparison of postoperative cognitive function outcomes.

Parameter	Group	D0 (baseline)	D3 (Postop day 3)	D7 (Postop day 7)	Mean difference [95% CI] (vs. Propofol)
MMSE Score (mean ± SD)	Remazolam	28.5 ± 1.2	28.0 ± 1.1*	28.3 ± 1.0*	
	Propofol	28.3 ± 1.4	26.8 ± 1.5	27.2 ± 1.3	*D3:* 1.20 [0.58, 1.82] *D7:* 1.10 [0.45, 1.75]
MoCA Score (mean ± SD)	Remazolam	27.9 ± 1.3	27.5 ± 1.2*	27.8 ± 1.1*	
	Propofol	27.7 ± 1.5	26.1 ± 1.6	26.5 ± 1.4	*D3:* 1.40 [0.71, 2.09] *D7:* 1.30 [0.62, 1.98]
POCD Incidence, n (%) (vs. Propofol)	Remazolam	—	3 (5.4%)**	2 (3.6%)***	
	Propofol	—	11 (19.6%)	8 (14.3%)	
POD Incidence (cumulative), n (%) (vs. Propofol)	Remazolam	—	1 (1.8%)	2 (3.6%)****	
	Propofol	—	6 (10.7%)	9 (16.1%)	

Data are mean ± SD, or n (%). MMSE, Mini-Mental State Examination; MoCA, montreal cognitive assessment; POCD, postoperative cognitive dysfunction; POD: postoperative delirium.

*P < 0.001 Remazolam group vs. Propofol group at the respective time point (Repeated Measures ANOVA, with Bonferroni *post hoc* test).

**P = 0.025 Remazolam group vs. Propofol group (Chi-square test).

***P = 0.048 for POCD, at D7 (Fisher’s exact test).

****P = 0.031 for POD, at D7 (Fisher’s exact test).

### 3.5 Adverse cardiovascular events

The overall incidence of intraoperative and early postoperative adverse cardiovascular events was significantly lower in the Remazolam group (21.4%) compared to the Propofol group (71.4%; P < 0.001) (Table 5). Specifically, the Remazolam group experienced significantly less hypotension (7.1% vs. 62.5%, P < 0.001) and bradycardia (14.3% vs. 53.6%, P < 0.001). There were no significant differences in the incidence of hypertension, tachycardia, or hypoxemia between the groups.

**TABLE 5:** Incidence of adverse cardiovascular events [n (%)].

Adverse event	Remazolam group (n = 56)	Propofol group (n = 56)	P-value
Hypotension	4 (7.1%)	35 (62.5%)	<0.001
Bradycardia	8 (14.3%)	30 (53.6%)	<0.001
Hypertension	3 (5.4%)	5 (8.9%)	0.465
Tachycardia	2 (3.6%)	4 (7.1%)	0.405
Hypoxemia (SpO_2_ <90%)	0 (0.0%)	2 (3.6%)	0.154
Any Cardiovascular Event (Overall)	12 (21.4%)	40 (71.4%)	<0.001

Data are n (%). P-values derived from Chi-square test or Fisher’s exact test.

### 3.6 Postoperative Analgesia and sedation

Patients in the Remazolam group reported significantly lower VAS pain scores at 6 and 12 h postoperatively (Table 6, all P < 0.001). Ramsay sedation scores were also significantly lower (indicating more alertness) in the Remazolam group at 6 and 12 h (all P < 0.001). The requirement for rescue analgesia was lower in the Remazolam group (10.7% vs. 28.6%, P = 0.018). The incidence of PONV was similar between groups (P = 0.537). The total intraoperative alfentanil consumption was comparable between the two groups (1.25 ± 0.31 mg in the Remazolam group vs. 1.29 ± 0.38 mg in the Propofol group, P = 0.582), suggesting that the observed differences in outcomes were not confounded by opioid dosage.

**TABLE 6:** Postoperative Analgesic and Sedative Assessment (mean ± SD or n [%]).

Parameter	Time post-surgery	Remazolam group (n = 56)	Propofol group (n = 56)	P-value
VAS Score (0–10)	6 h	1.78 ± 0.19	3.66 ± 0.42	<0.001
	12 h	1.15 ± 0.20	1.85 ± 0.52	<0.001
Ramsay Score (1–6)	6 h	1.82 ± 0.40	2.31 ± 0.28	<0.001
	12 h	1.21 ± 0.25	1.82 ± 0.40	<0.001
Rescue Analgesia (24 h)	—	6 (10.7%)	16 (28.6%)	0.018
PONV (24 h)	—	5 (8.9%)	7 (12.5%)	0.537

VAS, visual analog scale; PONV, Postoperative Nausea and Vomiting. Data are mean ± SD, or n (%). P-values derived from t-test for continuous variables and Chi-square test for categorical variables.

## 4 Discussion

This randomized controlled trial demonstrated that in elderly patients undergoing laparoscopic cholecystectomy, general anesthesia with a remazolam-alfentanil combination resulted in faster anesthesia recovery, superior intraoperative hemodynamic stability, better preservation of early postoperative cognitive function, and a lower incidence of adverse cardiovascular events compared to a propofol-alfentanil regimen. Additionally, the remazolam group reported lower pain scores and required less rescue analgesia postoperatively.

The rapid recovery profile observed with remazolam is consistent with its pharmacokinetic properties, namely, its rapid metabolism by tissue esterases to an inactive metabolite, leading to minimal accumulation and a short context-sensitive half-time (Kilpatrick, 2021; Zhou et al., 2020). Our findings align with previous studies showing shorter recovery times with remazolam compared to propofol in various procedural sedations and general anesthesia settings (Guo et al., 2022; Chen et al., 2020; Zhang et al., 2022). This rapid recovery can be particularly beneficial in elderly patients, potentially facilitating earlier mobilization and reducing PACU stay, as observed in our study.

Hemodynamic stability is a critical concern in elderly surgical patients. Propofol is known to cause dose-dependent vasodilation and myocardial depression, leading to hypotension and bradycardia (Chan and Chung, 1996). In contrast, remazolam has been reported to exert minimal effects on the cardiovascular system (Guo et al., 2022). Our study confirmed this, with the Remazolam group exhibiting significantly more stable MAP, HR, CO, and CI, and a markedly lower incidence of hypotension and bradycardia. This highlights remazolam’s potential as a safer alternative for maintaining hemodynamic stability in this vulnerable population, consistent with findings by other researchers (Tang et al., 2021; Qiu et al., 2022).

POCD and POD are common complications in elderly patients, associated with increased morbidity and prolonged hospital stays (Vutskits and Xie, 2016; Evered et al., 2018). The etiology is multifactorial, involving neuroinflammation, anesthetic neurotoxicity, and cerebral hypoperfusion (Berger et al., 2018). Our results indicate that patients in the Remazolam group had significantly better MMSE and MoCA scores and a lower incidence of both POCD and POD. These findings highlight a significant advantage in early cognitive recovery, although it is important to acknowledge that our assessment was limited to the first postoperative week, and longer-term cognitive trajectories were not evaluated. While a universally accepted minimal clinically important difference (MCID) for MMSE/MoCA in this specific postoperative elderly population is not definitively established, changes of 1-2 points on the MMSE are often considered clinically relevant (Tsoi et al., 2015). The observed mean differences between groups, particularly on day 7 (1.1 points for MMSE and 1.3 points for MoCA), therefore suggest a clinically meaningful advantage in early cognitive recovery for the remazolam group. The neuroprotective effect might be attributed to remazolam’s potential to attenuate neuroinflammation (Shi et al., 2022; Liu et al., 2021) and its more favorable hemodynamic profile, ensuring better cerebral perfusion. The more stable hemodynamics may prevent episodes of cerebral hypoperfusion, a known risk factor for POCD and POD (Suraarunsumrit et al., 2024; Wang et al., 2024).

The improved postoperative pain scores in the Remazolam group are intriguing. While remazolam itself is not an analgesic, its combination with a comparable dose of alfentanil might lead to a more stable anesthetic state, potentially modulating the stress response better than propofol-alfentanil and contributing to smoother emergence and better early pain control (Kramer et al., 1983; Xin et al., 2022).

This study has several strengths, including its prospective, randomized, and controlled design. However, some limitations should be acknowledged. First, this was a single-center study, which may limit generalizability. Second, while patients and outcome assessors were blinded, the administering anesthesiologist was not, which could introduce performance bias. Third, as mentioned, cognitive assessments were limited to the early postoperative period. Longer-term follow-up is needed. Fourth, the mechanisms underlying the better cognitive outcomes were not investigated at a molecular level (e.g., inflammatory markers).

## 5 Conclusion

In conclusion, for elderly patients undergoing laparoscopic cholecystectomy, general anesthesia with a remazolam-alfentanil combination offers significant advantages over propofol-alfentanil, including faster recovery, enhanced intraoperative hemodynamic stability, better preservation of early postoperative cognitive function, and a reduced incidence of adverse cardiovascular events. These findings suggest that remazolam combined with alfentanil may be a safer and more effective anesthetic option for this vulnerable patient population, contributing to an improved perioperative course. However, while promising, these single-center findings warrant confirmation in larger, multicenter trials with longer-term follow-up before definitive changes to clinical practice can be recommended.

## Data Availability

The original contributions presented in the study are included in the article/supplementary material, further inquiries can be directed to the corresponding authors.
